# Epigenetics of the preferential silencing of *Brachypodium stacei*-originated 35S rDNA loci in the allotetraploid grass *Brachypodium hybridum*

**DOI:** 10.1038/s41598-017-05413-x

**Published:** 2017-07-13

**Authors:** Natalia Borowska-Zuchowska, Robert Hasterok

**Affiliations:** Department of Plant Anatomy and Cytology, Faculty of Biology and Environmental Protection, University of Silesia in Katowice, 28 Jagiellonska Street, 40-032 Katowice, Poland

## Abstract

Nucleolar dominance (ND), initially described as ‘differential amphiplasty’, is a phenomenon observed in some plant and animal allopolyploids and hybrids in which the selective suppression of the activity of 35S rRNA gene loci that have been inherited from one of the two or more ancestral genomes occurs. Although more than 80 years have passed since the discovery of ND, there is still a significant lack in our understanding of the mechanisms that determine this phenomenon. Here, we aimed to investigate the epigenetic status of 35S rRNA gene loci in the monocotyledonous *Brachypodium hybridum*, which is an allotetraploid that has resulted from a cross between *B. distachyon* and *B. stacei*. We revealed that the repressed *B. stacei*-inherited rDNA loci are characterised by a high level of DNA methylation. The global hypomethylation of *B. hybridum* nuclear DNA induced by 5-azacytidine, however, seems to be insufficient for the transcriptional reactivation of these loci, which indicates that factors other than DNA methylation are behind the suppression of *B. stacei*-originated loci. We also showed that the transcriptionally active and silenced fractions of rRNA genes that had been inherited from *B. distachyon* occupy different domains within the chromocentres adjacent to the nucleolus, depending on their epigenetic status.

## Introduction

Allopolyploidisation consists of interspecific or intergeneric hybridisation, which is accompanied by chromosome doubling and is considered to be a prominent force in plant evolution^1, 2^. The genomes of newly formed natural allopolyploids, as well as resynthesised ones, undergo rapid changes at both the genetic and epigenetic levels, including chromosomal rearrangements, sequence loss, transposon proliferation, altered patterns of gene expression and meiotic irregularities^3–6^. In the long-term evolutionary time scale, however, the combination of two divergent genomes may lead to increased phenotypic variability. Such a variability of allopolyploids might be advantageous compared with their diploid parents, especially in the case of the rapid adaptation of allopolyploids to changed environmental conditions, which, therefore, may allow them to reach new environmental niches^7^.

Epigenetics refers to the heritable alterations of gene expression patterns without an underlying change in the DNA sequence^8^. It was shown that epigenetic modifications, e.g. DNA methylation and post-translational histone modifications, are among the factors that are behind the homoeologous gene expression changes in allopolyploids^6, 9–11^. The tandemly repeated 35S rRNA genes are of special interest in terms of the enigmatic phenomenon that is observed in hybrids and allopolyploids, which is called nucleolar dominance (ND), in which the nucleoli are formed in association with the 35S rRNA genes that had been inherited from one of two or more ancestors^12^. At least one example of intraspecific ND has been described to date^13, 14^. In the barley translocation line, which comprises NOR regions that originally belonged to chromosomes 6H and 7H and then occurred in one chromosome, the 35S rDNA loci from the 6H chromosome were dominant over the 7H ones^13–15^. Interestingly, it was shown that the role of DNA methylation in the suppression of 7H NOR is of limited importance^13^. In the case of interspecific or intergeneric ND, however, preferentially silenced rDNA loci can be reactivated by chemical inhibitors of DNA methylation (e.g. 5-azacytidine; 5-AzaC and 5-aza-2′-deoxycytidine) and histone deacetylation (trichostatin A), thus implicating the involvement of epigenetic mechanisms in ND maintenance^16–18^. Studies on ND in the allotetraploid *Arabidopsis suecica* revealed that the 35S rRNA gene promoter regions, which were inherited from both ancestors, differed in their 5-methylcytosine (5-MeC) content and modifications of the core histones. The under-dominant rDNA, which had been inherited from *A. thaliana*, was hypermethylated and enriched in the heterochromatic marker – dimethylated lysine 9 of histone H3 (H3K9me2), whereas the part of the dominant rDNA, which had originated from *A. arenosa*, was characterised by a low 5-MeC content and the presence of markers that are typical for euchromatin, e.g. trimethylated histone H3 at lysine 4 (H3K4me3), acetylated histone H3 at lysine 9 (H3K9ac) and hyperacetylated histone H4^19, 20^. Hence, concerted changes in the DNA methylation level and core histone modifications at the rDNA promoter region regulate the on and off states of the rRNA genes. Further investigations revealed that the components of the RNA-dependent DNA methylation pathway (RdDM), including RNA-dependent RNA polymerase 2 (RDR2), Dicer-like 3 (DCL3) and the DRM2 cytosine *de novo* methyltransferase, are implicated in ND enforcement^21, 22^. Although the understanding of the epigenetic mechanisms that are involved in ND has advanced significantly in the last decade, the question of how one set of rRNA genes is selected to be silenced in the allopolyploid organisms still needs to be answered.

In 2008, the occurrence of ND was revealed in the root meristematic cells of *Brachypodium hybridum* (2n = 30), which is an allotetraploid that has resulted from a cross between two putative ancestors that resembled modern *B. distachyon* (2n = 10) and *B. stacei* (2n = 20)^23^. Our recent studies showed that the preferential inactivation of *B. stacei*-inherited 35S rDNA in the allotetraploid is also present in the nuclei of differentiated root cells. An analysis of 35S rDNA intergenic spacers (IGSs) from both *B. hybridum* and its putative ancestors suggested that although some genetic changes in the sequence of the under-dominant *B. stacei*-like rDNA might be behind the selective inactivation of the aforementioned loci, the involvement of reversible, epigenetic mechanisms cannot be excluded^24^.

The aim of the presented paper was to verify whether DNA methylation and post-translational modifications of the core histones are involved in the preferential silencing of *B. stacei*-inherited 35S rDNA in *B. hybridum*. An immunostaining assay using antibodies raised against 5-MeC was used to determine whether there are any differences in the 5-MeC content between the 35S rDNA loci that had been inherited from both ancestors. The transcriptional activity of these loci was verified using the silver staining method after the 5-AzaC treatment in order to determine the effect of DNA methylation on the preferential inactivation of rRNA genes in *B. hybridum*. Moreover, the immunopatterns of several histone modifications (H3K9me2, H3K9ac, H4K5ac and H4K16ac), which are potentially involved in ND enforcement, were determined in the interphase nuclei of the studied allotetraploid.

## Results and Disscussion

### DNA methylation is involved in the selective inactivation of rRNA genes in *B. hybridum*

**Figure 1:**
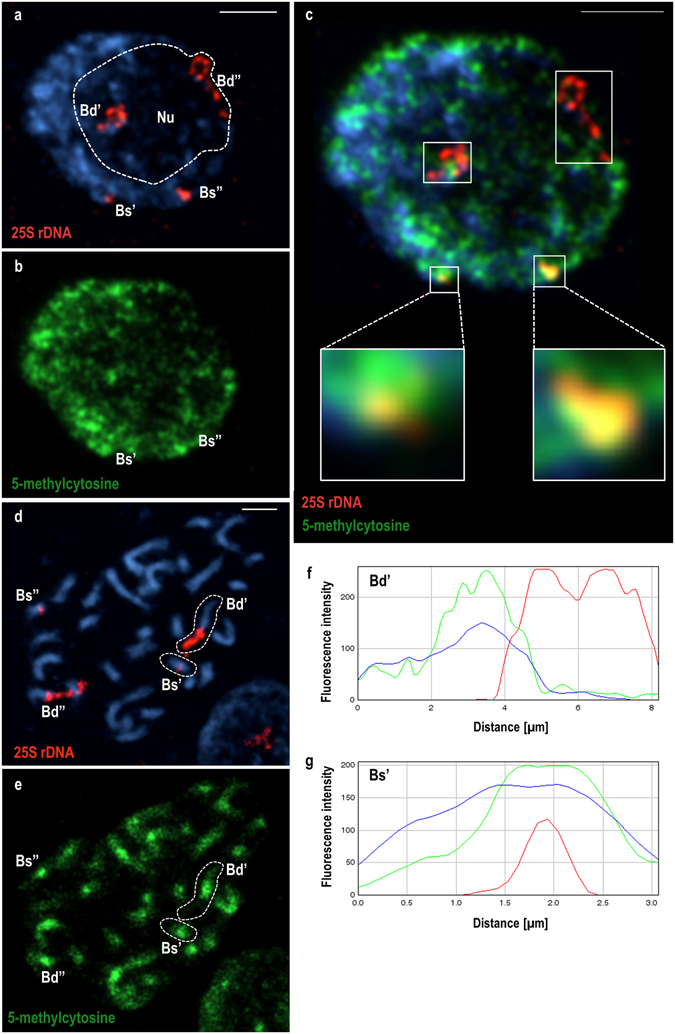
DNA methylation patterns of 35S rRNA genes in *B. hybridum*. **(a)** FISH with 25S rDNA (red fluorescence) as a probe in the representative interphase nucleus of *B. hybridum* (genotype ABR113). **(b)** 5-MeC immunopattern (green fluorescence) in the same nucleus. (**c)** Superimposed images of nucleus after FISH with 25S rDNA **(a)** and signals of 5-MeC residues **(b)**. *B. stacei*-inherited 35S rDNA loci are enlarged in the insets. **(d)** FISH with 25S rDNA (red fluorescence) as a probe in the mitotic metaphase chromosomes of *B. hybridum* (genotype ABR101). **(e)** 5-MeC foci distribution in the metaphase complement shown on **(d)**. **(f,g)** 5-MeC foci distribution along the longitudinal axes of chromosomes Bd’ **(f)** and Bs’ **(g)** which are surrounded by dashed line on **(d,e)**. The blue curves denote the fluorescence intensity of the counterstain (DAPI), the green curves indicate the distribution of methylation foci. The position of the 35S rDNA loci is shown by the red curves. The length of chromosomes is shown on the *x-*axis in microns, while the fluorescence intensity on the *y-*axis is presented in arbitrary units. Nu – nucleolus. Scale bar: 5 µm.

The methylation of cytosine residues in DNA is among the epigenetic marks that have a great impact on gene expression patterns through downregulation when it concerns the promoter regions, or sometimes upregulation via the methylation of the gene bodies^25–27^. It is well known that DNA methylation plays a central role in the maintenance of ND in many plant allopolyploids^16, 28^. In the case of *B. hybridum*, however, the involvement of this epigenetic mark in the preferential suppression of rRNA genes has not yet been tested. For this reason, the DNA methylation patterns of 35S rDNA loci in three *B. hybridum* genotypes (ABR101, ABR113 and ABR137) were investigated via the immunodetection of 5-MeC, followed by FISH with 25S rDNA as a probe. The distribution of the 5-MeC foci was studied in both the interphase nuclei (Fig. 1a–c) and mitotic metaphase chromosomes (Fig. 1d–g) from *B. hybridum* root-tips. At least 30 nuclei and 30 metaphase spreads were taken into account for each of the studied genotypes. In *B. hybridum* metaphase spreads, the 35S rDNA loci inherited from both ancestors can be discriminated based on their position in the chromosome. It was shown that *B. distachyon-*inherited loci occupy the distal parts of the short arms of one chromosome pair, while *B. stacei-*originated loci are located more proximally in the other, significantly smaller chromosome pair^23^. At the level of interphase nuclei, additional markers such as species-specific intergenic spacers (IGSs) and chromosome-specific BAC clones are required to differentiate these loci. It was documented by FISH with either chromosome-specific BACs or IGSs along with 25S rDNA as probes that only *B. distachyon*-like loci associate with the nucleolus in the *B. hybridum* nuclei isolated from roots^24^.

As can be seen in Fig. 1, significant differences in the DNA methylation levels were observed between the rDNA loci that had been inherited from both ancestors. In the interphase nuclei, the *B. distachyon*-inherited 35S rDNA loci that were associated with the nucleolus were characterised by a lower density of 5-MeC foci compared with the peripherally located *B. stacei-*inherited rDNA sites (Fig. 1a–c). The 25S rDNA hybridisation signals that corresponded with the *B. stacei-*like loci colocalised with strong anti-5-MeC signals (Fig. 1c and insets therein). In the case of the metaphase chromosomes, the anti-5-MeC signals were additionally measured along the longitudinal axes of the 35S rDNA-bearing chromosomes. The representative methylation profiles for the *B. distachyon-* (Bd) and *B. stacei-*inherited (Bs) chromosomes with these rRNA gene loci are shown in Fig. 1f and g, respectively. As was expected, the 25S rDNA hybridisation signal (red curve; Fig. 1f) in the *B. distachyon-*like chromosomes colocalised at a low DNA methylation level (green curve; Fig. 1f), which reflected the transcriptional activity of these loci. In contrast, the proximally located *B. stacei-*inherited rDNA loci were characterised by a high density of 5-MeC foci (Fig. 1g). Such a distribution of anti-5-MeC signals within the studied loci strongly suggests that DNA methylation is involved in the transcriptional repression of *B. stacei-*inherited rDNA in *B. hybridum*.

Similar differences in the 5-MeC content between the dominant and repressed 35S rDNA were observed in dicot allotetraploids belonging to the *Brassica* ^16^ and *Arabidopsis* ^20–22^ genera and some monocot representatives including cereal allopolyploids^29, 30^. The rDNA hypermethylation, which concerns the promoter sequences of rRNA genes, is always correlated with their transcriptional inactivation. In triticale, for instance, the presence of wheat 1B and 6B chromosomes, which contain the dominant 35S rDNA loci, enhanced the methylation of the rRNA gene promoters that were of rye origin. Interestingly, the deletion of the wheat 1B chromosome led to an observable decrease in the DNA methylation level of the rye rDNA and, as a result, to an increase in the transcriptional activity of rye NORs^29^. Moreover, studies on wheat-rye addition lines have shown that the repression of rye-originated rDNA also correlated with an increase in the 35S rDNA activity in wheat chromosomes, thus the rRNA gene expression changes in ND encompass both the dominant and under-dominant loci^31^. Collectively, these data indicate that DNA methylation plays a key role in the regulation of the rRNA gene dosage control and ND enforcement.

### Global demethylation of the DNA caused by 5-AzaC appears to be insufficient for the transcriptional reactivation of *B. stacei*-inherited rDNA

**Figure 2:**
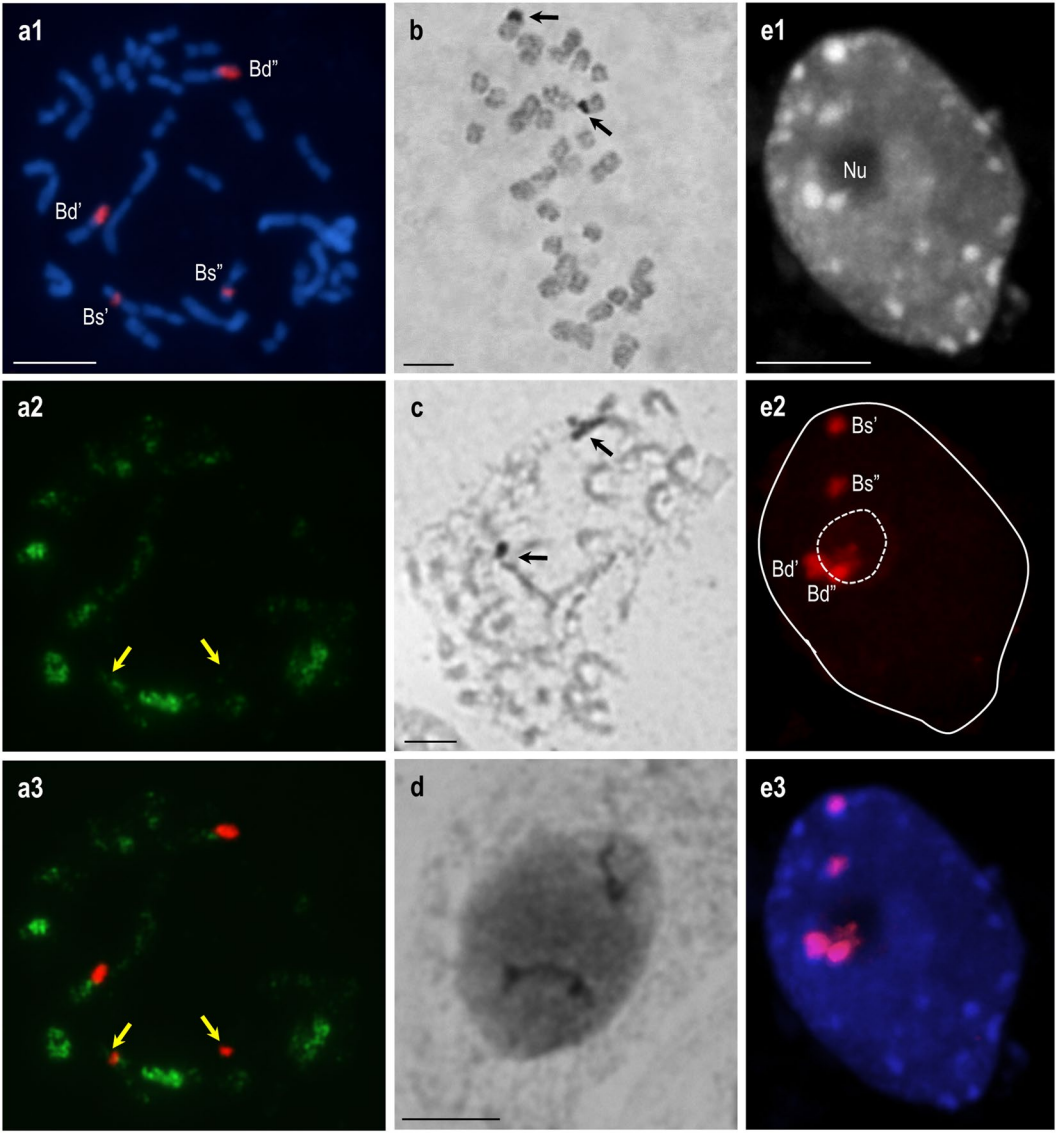
Impact of DNA hypomethylation caused by 0.1 mmol/L 5-AzaC on the preferential inactivation of 35S rRNA genes in the metaphase plates **(a1–a3**; **c)** and nuclei **(d**; **e1–e3)** of *B. hybridum* (genotype ABR113). The 5-AzaC treatment changed neither the number of silver-stained loci, nor the non-nucleolar position of *B. stacei*-inherited 35S rDNA loci. **(a1)** FISH with 25S rDNA (red fluorescence) as a probe in the mitotic metaphase chromosomes. **(a2)** 5-MeC foci distribution in the metaphase spread shown on **(a1**). (**a3)** Superimposed images of 25S rDNA FISH signals and anti-5-MeC signals. *B. stacei-*inherited loci are highlighted by yellow arrows. **(b–d)** The activity of 18S-5.8S-25S rDNA loci in the control (i.e. not treated with 5-AzaC) root-tip cells **(b)** and after 0.1 mmol/L 5-AzaC treatment in the metaphase plate **(c)** and interphase nucleus **(d)**. The Ag-NOR bands are highlighted by black arrows. **(e1)** DAPI counterstained nucleus. **(e2)** 25S rDNA hybridisation signals (red fluorescence). **(e3)** Superimposed images of 25S rDNA FISH signals and counterstained chromatin. Nu–nucleolus. Scale bar: 5 µm.

It is well documented that rRNA gene loci repression via ND is a completely reversible, developmentally regulated process. In the case of the allotetraploid *B. napus*, ND was observed in the vegetative tissues^32^ with the exception of the root-tip cells^33^. Interestingly, the vegetative to generative transition in this allotetraploid was accompanied by activation of the under-dominant rRNA gene loci that was set in all of the floral organs, including sepals and petals^32^. It was shown that the transition from the vegetative to generative phase is accompanied by a significant decrease in the DNA methylation level and a higher level of acetylated histone H4^34, 35^. Taking this data into account, the activation of the under-dominant rDNA loci was most probably associated with changes at the epigenetic level^32^. It was confirmed that the inhibition of either DNA methylation or histone deacetylation in allopolyploids that exhibit ND resulted in the derepression of rRNA gene loci that had previously been silenced^16, 17, 20, 36^. To determine whether global demethylation of the *B. hybridum* genome has an effect on selective suppression of *B. stacei-*originated rRNA gene loci, treatment with a hypomethylating agent, 5-AzaC, was performed during seed germination. The demethylating effect of 5-AzaC at a concentration of 0.1 mmol/L has previously been tested on the root-tip cells of *B. distachyon* and it was shown that application of this drug led to loss of recurrent methylation profiles in NOR chromosomes^37^. A similar situation was observed in the root-tip cells of *B. hybridum* after 5-AzaC treatment, wherein the hypomethylation of *B. stacei-*like 35S rDNA loci was observed, as demonstrated by sequential immunodetection of 5-MeC and FISH with 25S rDNA as a probe (Fig. 2a1–a3). The activity of 35S rDNA loci was verified using the silver staining procedure. As can be seen in Fig. 2b, in an untreated, control root-tip metaphase plate from *B. hybridum* (genotype ABR113), only the rDNA-containing chromosome pair that had been inherited from *B. distachyon* bore Ag-NOR bands, as was shown previously by sequential silver staining and FISH^23^. Interestingly, the number of sites that were stained by silver was not changed on both the metaphase spreads (Fig. 2c) and interphase nuclei (Fig. 2d) after the 5-AzaC treatment. The inactive state of *B. stacei-*inherited loci was also confirmed at the level of interphase nuclei isolated from 5-AzaC-treated *B. hybridum* roots. It was shown that the aforementioned loci were located at the nuclear periphery and did not associate with the nucleolus (Fig. 2e1–e3), which strongly suggests that mechanisms other than DNA methylation are also involved in the repression of *B. stacei-*inherited rRNA genes in *B. hybridum*. A similar effect was observed in diploid *Quercus robur* root-tip cells. After 5-AzaC treatment, no additional Ag-positive bands were found in the metaphase spreads, suggesting that other epigenetic modifications or changes at the genetic level may be involved in the 35S rDNA loci inactivation in this species^38^.

Based on the results obtained in this study and our molecular analysis of the *B. hybridum* intergenic spacers^24^, we can speculate that *B. stacei-*inherited 35S rRNA genes have lost their function and have evolved as pseudogenes. In our previous study, only the *B. distachyon*-like intergenic spacer (IGS) of rRNA genes was amplified in *B. hybridum*. Such an amplification pattern may be connected with a gradual accumulation of mutations in the inactive rDNA loci, which involved a conserved primer binding sites in 18S and 25S rDNA sequences^24^. Moreover, in the case of at least one *B. hybridum* genotype (ABR117), the presence of only *B. distachyon-*like rDNA loci has been demonstrated^24, 39^, which may corroborate the progressive elimination of repressed rDNA loci in this allotetraploid^24^. This hypothesis, however, still requires stronger support through transcriptome analyses.

### *B. distachyon-* and *B. stacei-*inherited 35S rDNA loci in *B. hybridum* are characterised by distinct post-translational histone modifications

Apart from their role as structural components of chromatin, histones are involved in the regulation of gene expression^40^. It is well known that particular modifications of the N- and C-terminal histone tails can be attributed to a transcriptionally active or silent state of the coding sequences. Whereas the acetylated lysines of histones H3 and H4 were found to be associated with actively transcribed genes, the methylated ones correlated with both up- and downregulated genes depending on which residue in the histone tail was modified^41^. The influence of the histone acetylation and methylation in the regulation of rRNA gene expression has been well documented^19, 20, 38^.

**Figure 3:**
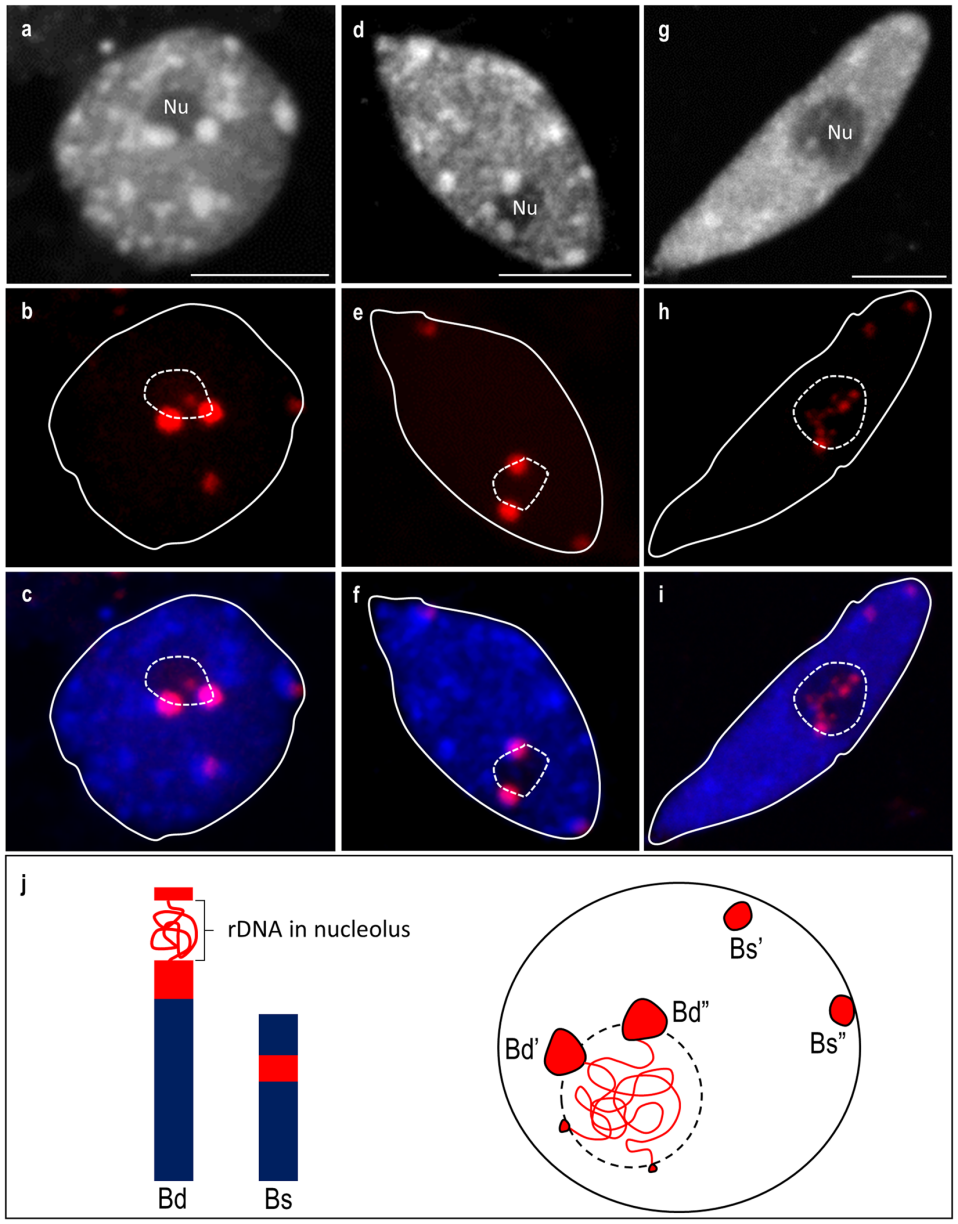
Distribution of 25S rDNA FISH signals in *B. hybridum* root nuclei of different shapes: spherical **(a–c)**, elongated **(d–f)** and rod-shaped **(g–i)**. **(a**,**d**,**g)** DAPI counterstained nuclei. **(b**,**e**,**h)** 25S rDNA hybridisation signals (red fluorescence). **(c**,**f**,**i)** Superimposed images of 25S rDNA FISH signals and counterstained chromatin. **(j)** The model of the 35S rRNA genes distribution in metaphase chromosomes (left) and interphase nucleus (right) of *B. hybridum*. Nu–nucleolus. Scale bar: 5 µm.

In the present study, the determination of the epigenetic status of 35S rRNA gene loci in interphase nuclei isolated from *B. hybridum* roots consisted of two steps: (i) determination of the positions of the loci that had been inherited from both ancestral species and (ii) establishment of the immunopatterns of different histone modifications characteristic for eu- or heterochromatin. Although the shapes of the nuclei that had been isolated from *B. hybridum* roots varied from spherical (Fig. 3a–c) through elongated (Fig. 3d–f) to rod-like ones (Fig. 3g–i), the first two types were the most common as was shown previously^42^. In order to investigate the distribution of rRNA gene loci, FISH with 25S rDNA as a probe was performed. It was found that in all types of nuclei, the *B. stacei*-inherited 35S rDNA loci were localised in the highly condensed, heterochromatic chromocentres at the nuclear periphery, whereas the loci that had been inherited from *B. distachyon* were associated with the nucleolus (Fig. 3). The *B. distachyon-*like 25S rDNA signals were present in either the chromocentres adjacent to the nucleolus (Fig. 3d–f) or within the nucleolus (Fig. 3a–c,g–i). As was shown for many plant species^19, 38, 43^, there is a strong correlation between the position of the 18S-5.8S-25S rDNA loci in the nucleus and their activity. Only actively transcribed rRNA genes associate with the nucleolus^38^. Based on the data obtained in this work and our previous studies^23, 24^, a model of 35S rDNA distribution at the chromosomal and nuclear level in *B. hybridum* was created (Fig. 3j), according to which only the rDNA loci that had been inherited from *B. distachyon* constituted decondensed nucleolar organiser regions (NORs).

**Figure 4:**
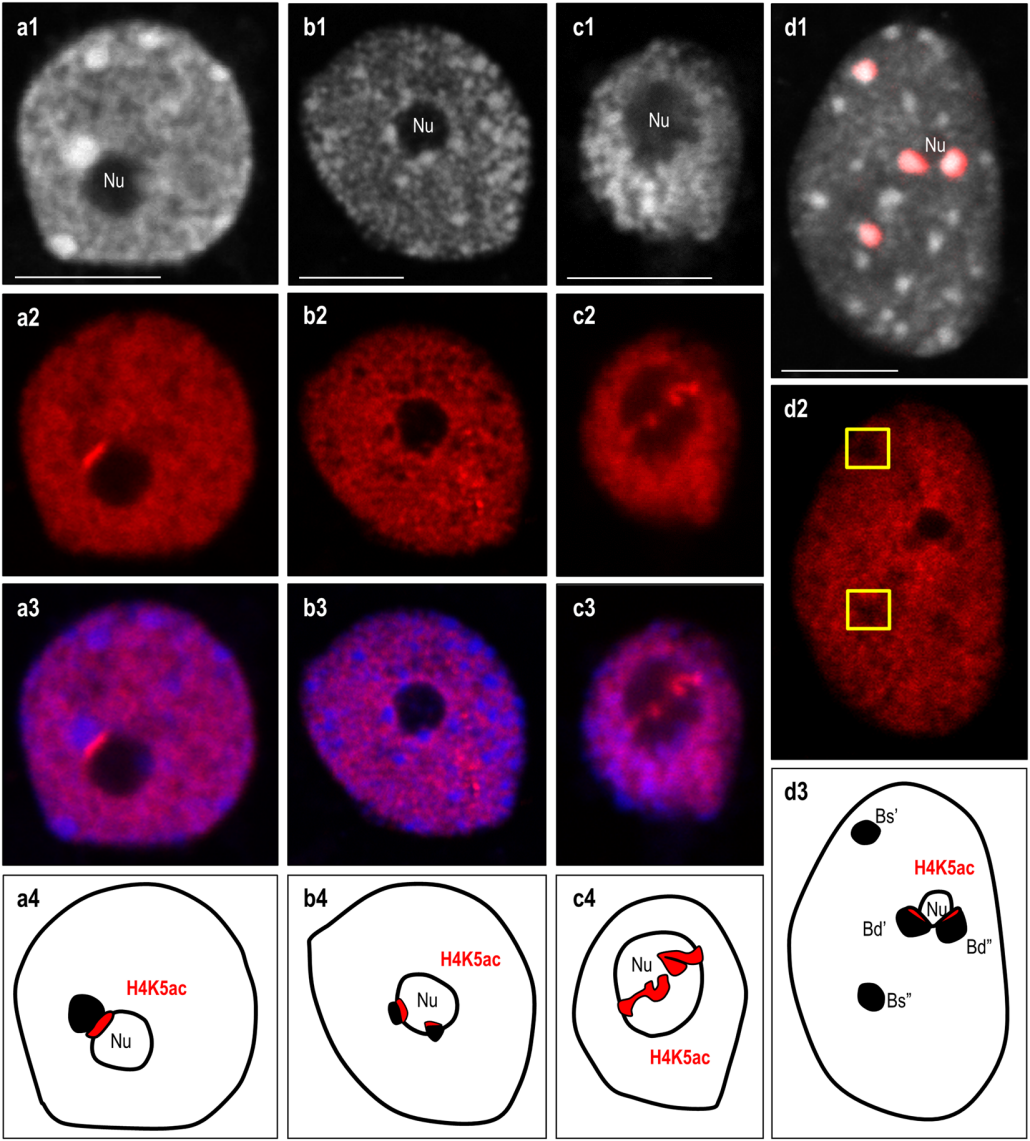
Immunopatterns of H4K5ac in the nuclei isolated from *B. hybridum* roots. H4K5ac foci distribution in nuclei with one **(a1–a4)**, two **(b1–b4)** or none **(c1–c4)** chromocentre(s) adjacent to the nucleolus. **a1**,**b1**,**c1**–DAPI counterstained nuclei; **a2**,**b2**,**c2**–H4K5ac foci distribution; **a3**,**b3**,**c3**–superimposed channels H4K5ac foci distribution (red) and DAPI (blue); **a4**,**b4**,**c4**–schematic representation of H4K5ac distribution in the nucleolus and adjacent chromocentres. **(d1)** FISH with 25S rDNA (red) as a probe in *B. hybridum* nucleus. **(d2)** H4K5ac immunostaining pattern in nucleus presented on **(d1)**. The locations of *B. stacei*-originated 35S rDNA loci are indicated by yellow insets. **(d3)** Schematic representation of H4K5ac foci distribution in the 35S rDNA loci superimposed from *B. distachyon* (Bd’; Bd”) and *B. stacei* (Bs’; Bs”) in *B. hybridum* nucleus. Nu – nucleolus. Scale bar: 5 µm.

**Figure 5:**
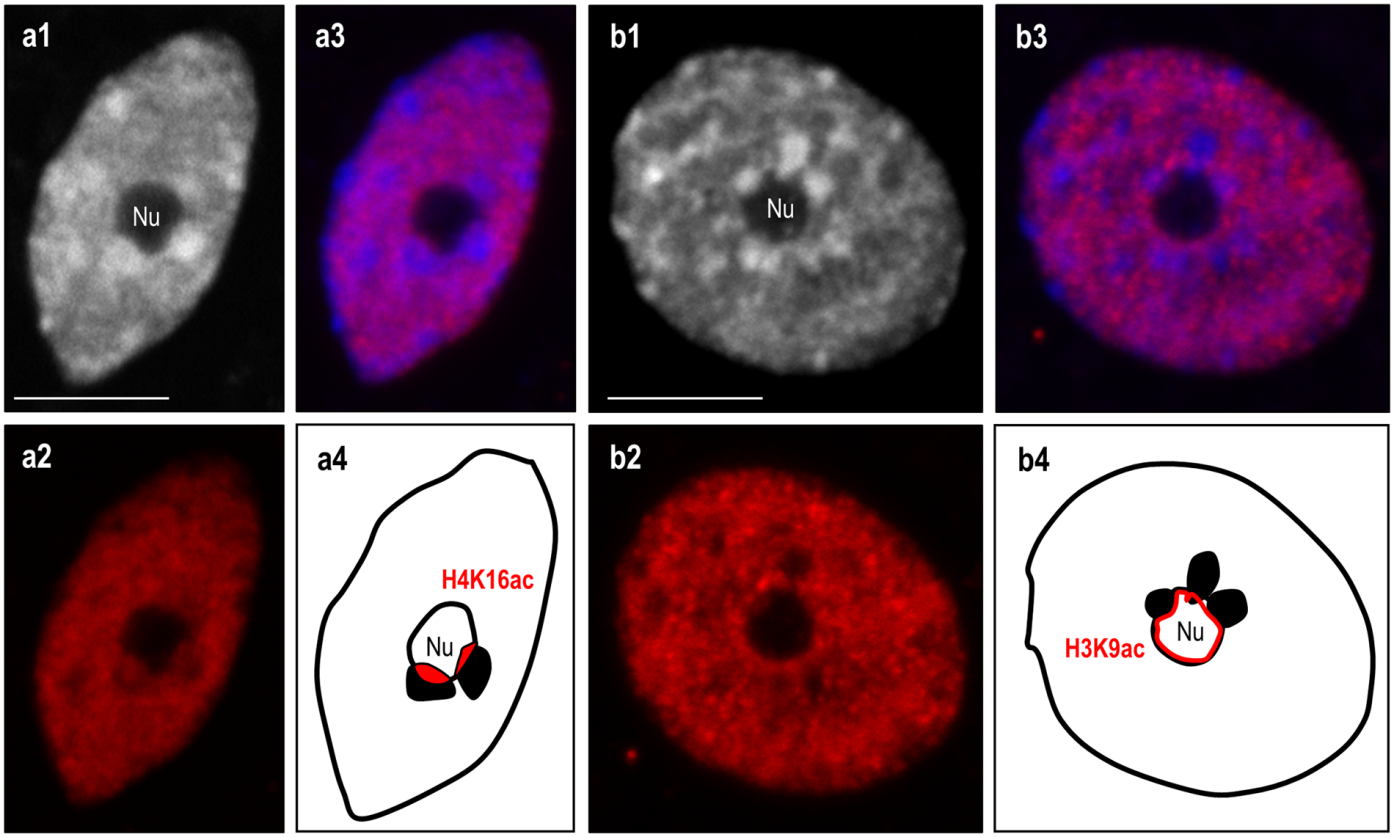
Immunopatterns of H4K16ac **(a1–a4)** and H3K9ac **(b1–b4)** in the nuclei isolated from *B. hybridum* roots. **a1**,**b1**–DAPI counterstained nuclei; **a2**,**b2**–histone immunopatterns; **a3**,**b3**–superimposed channels of particular histone modification foci distribution (red) and DAPI (blue); **a4**,**b4**–schematic representation of H4K16ac **(a4)** and H3K9ac **(b4)** foci distribution in the nucleolus and adjacent chromocentres. Nu – nucleolus. Scale bar: 5 µm.

**Figure 6:**
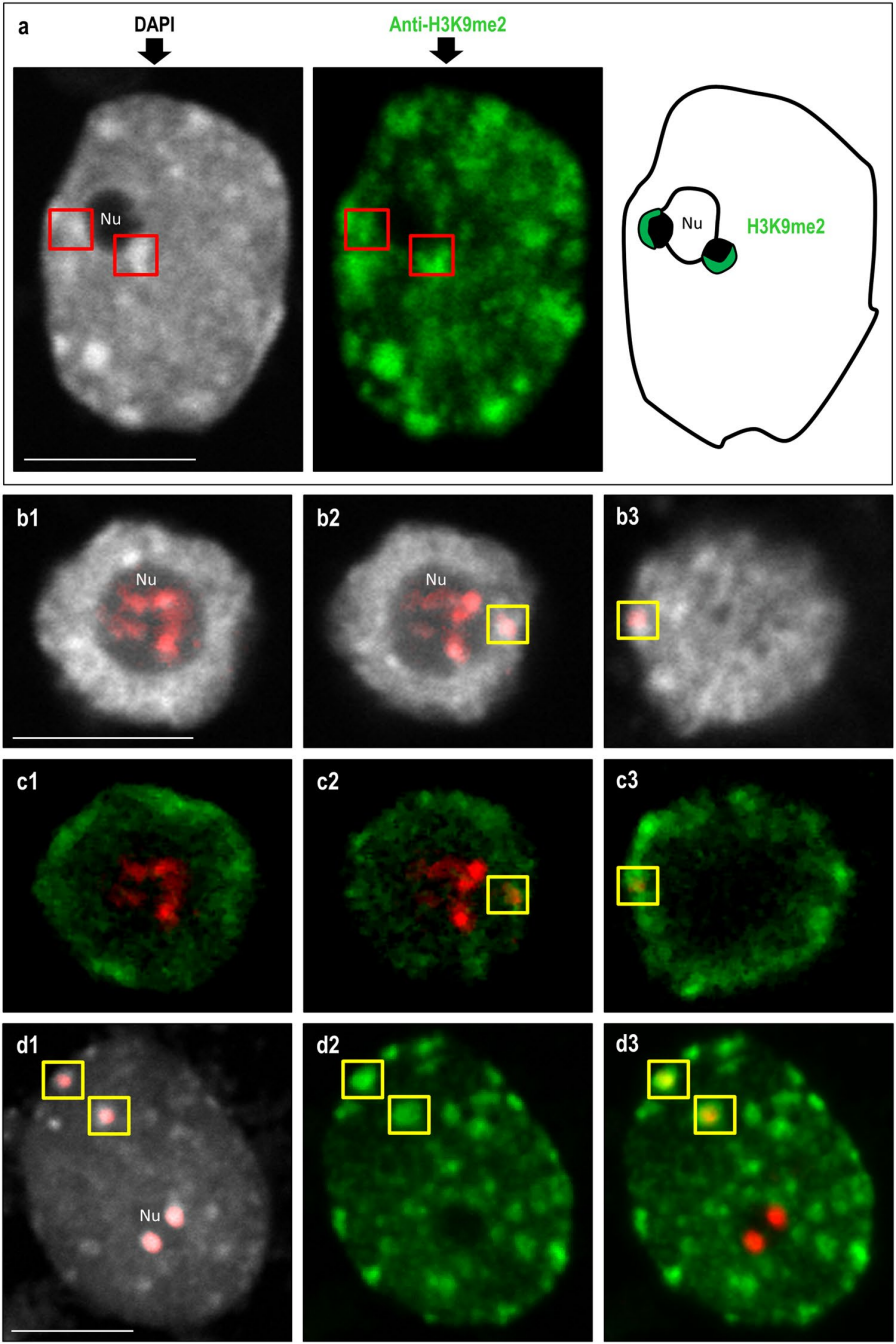
Distribution of heterochromatic marker H3K9me2 in the nuclei of *B. hybridum*. **(a)** H3K9me2 immunopattern in the chromocentres adjacent to the nucleolus. (**b1–b3**; **c1–c3**) H3K9me2 foci distribution in the nucleus without distinct heterochromatic domains. Three example sections through the representative nucleus are presented. **(b1–b3)** FISH with 25S rDNA (red) as a probe. **(c1–c3)** H3K9me2 foci (green) distribution and 25S rDNA FISH signals (red) in the sections through nucleus presented on **(b1–b3)**. **(d1–d3)** H3K9me2 foci distribution in the nucleus with chromocentres. **(d1)** FISH with 25S rDNA (red) as a probe. **(d2)** H3K9me2 foci distribution in the nucleus shown on **(d1)**. **(d3)** H3K9me2 foci (green) distribution and 25S rDNA hybridisation signals (red) in the nucleus presented on **(d1)**. The locations of *B. stacei*-originated 35S rDNA loci are indicated by yellow insets. Nu–nucleolus. Scale bar: 5 µm.

**Figure 7:**
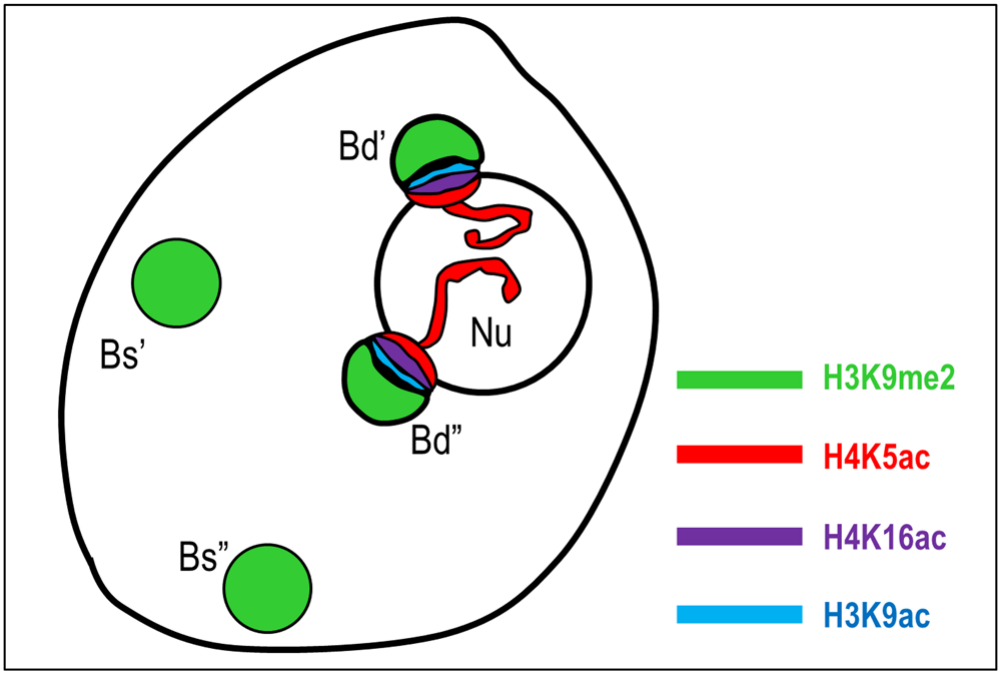
Model of active and silent 35S rDNA loci distribution and their epigenetic status in the nuclei of *B. hybridum*.

The immunopatterns of four different histone modifications that are linked to either euchromatin (H4K5ac; H4K16ac; H3K9ac) or heterochromatin (H3K9me2) were investigated in the interphase nuclei of the studied allotetraploid. Their involvement in the ND enforcement was previously documented in the dicotyledonous allotetraploid *A. suecica* ^19, 20^. In *B. hybridum* nuclei, the immunofluorescence signals corresponding to the acetylated isoforms of histones H3 and H4 were primarily distributed in the euchromatic regions (Figs 4 and 5). The immunostaining was absent in the DAPI-positive domains, with the exception of the chromocentres adjacent to the nucleolus. The most prominent immunofluorescence signals that corresponded with the acetylated histones H3 and H4 were detected at the chromocentre/nucleolus boundary, especially in the case of H4K5ac (Fig. 4). Such anti-H4K5ac signals were found in the nuclei with one (Fig. 4a1–a4) or more (Fig. 4b1–b4) chromocentres that lay next to the nucleolus. Interestingly, in nuclei that had no easily distinguishable heterochromatic domains, two strong immunofluorescence signals that corresponded with H4K5ac were also observed in the nucleolus area (Fig. 4c1–c4), which most probably reflects the active fraction of the 35S rRNA genes that had been inherited from *B. distachyon*. To verify the H4K5ac immunopattern in the *B. stacei* inherited rDNA loci, immunostaining with anti-H4K5ac antibodies followed by FISH with 25S rDNA as a probe was performed. The lack of this modification in the aforementioned loci was observed (Fig. 4d1–d3), which further confirmed their inactive status in the studied nuclei. Although the presence of both H4K16ac (Fig. 5a1–a4) and H3K9ac foci (Fig. 5b1–b4) was also confirmed at the boundary of the nucleolus, the immunofluorescence signals were not as prominent as those of the H4K5ac foci. In contrast, the anti-H3K9me2 signals were primarily present in the heterochromatic chromocentres with some signals of a low intensity in euchromatin (Fig. 6a). It is worth noting, however, that the chromocentres adjacent to the nucleolus were not uniformly marked by the H3K9me2 foci. Strong signals corresponding to H3K9me2 were present at the opposite poles of these chromocentres compared to the H4K5ac foci (Fig. 6a). Such a distribution of the acetylated isoforms of histones H3 and H4 and the heterochromatin landmark, H3K9me2, in the chromocentres adjacent to the nucleolus suggests that the actively transcribed and silenced fractions of rRNA genes occupy distinct domains in the nucleolus-associated chromocentres. The actively transcribed fraction, which is distributed at the chromocentre/nucleolus boundary as well as within the nucleolus, is associated with euchromatic markers: acetylated histones H3 (K9) and H4 (K5; K16), while the repressed rRNA genes are enriched by H3K9me2 and are localised at the opposite pool of the chromocentre (Fig. 7). A similar conclusion was drawn in the work of Pontvianne *et al*.^44^ for *A. thaliana hda6* and *met1* mutants showing that 35S rRNA genes present within the nucleolus were transcriptionally active, while those excluded from the nucleolus were repressed^44^. Studies on rye metaphase chromosomes revealed that the inactive fraction of rRNA genes is localised at the edge(s) of the secondary constrictions^45^. By contrast, within the NORs of yeast, active and silenced rRNA genes do not occupy distinct domains but are distributed randomly^46, 47^, suggesting a different regulation of rRNA gene expression in yeast and plants.

The H3K9me2 immunopattern was also determined in the *B. stacei*-like rDNA loci using sequential immunostaining method and FISH with 25S rDNA. It was shown that in nuclei without distinct heterochromatic domains (Fig. 6b1–b3,c1–c3) and nuclei with chromocentres (Fig. 6d1–d3), the *B. stacei*-originated loci were enriched by H3K9me2. The presence of this modification was also found in repressed rDNA loci of wheat^1^ and in the silenced *A. thaliana*-derived loci in allotetraploid *A. suecica* ^20^. These results suggest that the concerted DNA methylation (Fig. 1) and histone H3K9 dimethylation (Fig. 6) maintain the preferential silencing of the *B. stacei-*inherited loci. The fact that demethylation caused by a high dose of 5-AzaC is insufficient to reactivate the silenced rDNA loci along with the results of our previous analysis of rDNA intergenic spacers^24^ strongly suggest that genetic factors may be involved in the genome-specific rDNA loci suppression in *B. hybridum*.

## Materials and Methods

### Plant material and seed germination

Three different *B. hybridum* genotypes (ABR101 from South Africa, ABR113 from Portugal, ABR137 from Australia; all 2n = 30) were used in this study. Seeds were obtained from the Institute of Biological, Environmental and Rural Sciences (IBERS), Aberystwyth University, UK.

The dehusked seeds were grown on filter paper moistened with either tap water (control) or a 5-AzaC solution (0.1 mmol/L; Sigma-Aldrich) for 3 days at room temperature in the dark. The seeds were transferred onto a fresh 5-AzaC solution every day. Whole seedlings with two to three-cm-long roots were immersed in ice-cold water for 24 h, fixed in a 3:1 (v/v) methanol/glacial acetic acid at 4 °C overnight and stored at −20 °C until use.

### Root meristem squash preparation

Chromosome preparations were made as described^48^. Roots were washed in a 0.01 mmol/L citric acid – sodium citrate buffer (pH 4.8) and digested enzymatically for 1.5 h at 37 °C in a mixture comprising 2% (w/v) cellulase ‘Onozuka R-10’ (Serva) and 20% (v/v) pectinase (Sigma-Aldrich). After digestion, the meristems were dissected from the root tips and squashed in a drop of 45% acetic acid. After freezing on dry ice, the cover slips were removed and the slides were air dried.

### Preparation of nuclei

The immunopatterns of selected histone modifications were analysed in interphase nuclei that had been isolated from two-cm-long *B. hybridum* roots (genotype ABR113). The isolation was performed as described^24^. Briefly, 40 seedlings were fixed in 4% formaldehyde in 1 × PBS (pH 7.3) for 30 min at 4 °C and washed in 1 × PBS for 10 min. The separated roots were incubated in a Tris buffer at 4 °C for 20 min and then chopped in a 400 µL LB-01 buffer on an ice-cold Petri dish. The nuclei suspension was filtered through nylon mesh (30 µm pores) and approximately 30 µL of filtered suspension was dropped on each ice-cold slide and air-dried. The slides were stored at −20 °C until use.

### 5-methylcytosine immunodetection

The immunodetection of 5-MeC was performed as described previously^37^ using a mouse monoclonal antibody raised against 5-MeC (Abcam; 1:200 dilution in 1% BSA in 1 × PBS) and goat anti-mouse secondary antiserum conjugated with Alexa Fluor 488 (Invitrogen; 1:200 dilution in 1% BSA in 1 × PBS). Slides were denatured in 70% formamide at 70 °C for 2 min and blocked in 5% BSA. After the incubation with primary antiserum at 37 °C for 1 h, the slides were washed in 1 × PBS. The incubation with the secondary antibody was done at 37 °C for 1 h. The chromosomes and nuclei were counterstained with 2.5 µg/ml 4′,6-diamidino-2-phenylindole (DAPI; Serva) in Vectashield antifade buffer (Vector Laboratories).

### Immunodetection of post-translational histone modifications

The following mono- and polyclonal rabbit antisera raised against selected modifications of histones H3 and H4 were used: anti-H3K9me2 (Upstate), anti-H3K9ac (Abcam), anti-H4K5ac (Upstate) and anti-H4K16ac (Upstate). As secondary antibody, goat anti-rabbit IgG conjugated with Alexa Fluor 488 (Invitrogen) was used. All antibodies were diluted 1:100 in 1% BSA in 1 × PBS. Immunostaining was carried out as described^8^. The slides with isolated nuclei were blocked in 5% BSA and incubated with primary antiserum at 4 °C overnight. After washing in 1 × PBS, the secondary antibody was applied at 37 °C for 1 h. Nuclei were counterstained with DAPI in Vectashield.

### Fluorescence *in situ* hybridisation

A 2.3 kb fragment of the 25S rDNA of *A. thaliana*, which was labelled with tetramethyl-rhodamine-5-dUTP using nick translation, was used as the probe for fluorescence *in situ* hybridisation (FISH) to determine the position of the 35S rDNA loci in interphase nuclei and on mitotic metaphase chromosomes of *B. hybridum*. When FISH was performed after immunostaining with antibodies raised against 5-MeC, the slides were evaluated for the patterns of immunofluorescent signals and washed in 4 × saline sodium citrate (SSC) with 0.1% Tween 20 at 37 °C. Then, slides were washed briefly in 2 × SSC at room temperature, dehydrated in ethanol series and air-dried.

The FISH procedure was carried out as described previously^49^. The labelled 25S rDNA was precipitated and dissolved in a hybridisation mixture that consisted of 50% deionised formamide, 10% dextran sulphate and 2 × SSC buffer. The mixture was predenatured at 75 °C for 10 min and applied to the preparations with either the metaphase chromosomes after immunostaining or isolated nuclei from roots. The final denaturation step was performed at 75 °C for 4.5 min. After the hybridisation at 37 °C overnight, the post-hybridisation washes were performed in 10% deionised formamide in 0.1 × SSC for 10 min at 42 °C (corresponding to 79% stringency). Chromosomes and nuclei were counterstained with DAPI in Vectashield (Vector Laboratories).

In order to determine the epigenetic status of *B. stacei-*inherited 35S rDNA loci, an additional immunodetection with antibodies raised against H3K9me2 and H4K5ac, followed by FISH with 25S rDNA as a probe, was performed. Slides after the incubation with the secondary antibody were crosslinked in 4% formaldehyde in 1 × PBS (pH 7.3) at 4 °C for 10 min, washed twice in 2 × SSC for 10 min, dehydrated in an ethanol series and air-dried. The FISH reaction with 25S rDNA as a probe was performed on preparations without an initial evaluation for the patterns of immunofluorescent signals. The hybridisation mixture was applied to the preparations directly after cross-linking procedure.

### Image acquisition and processing

Photomicrographs of chromosomes and nuclei from squashed root meristems (sequential 5-MeC immunostaining and FISH) were taken using an AxioCam HRm monochromatic camera attached to a wide-field AxioImager.Z2 epifluorescence microscope (Zeiss). All images were processed using MBF ImageJ (NIH, US). To determine the 5-MeC immunopatterns of 35S rDNA-bearing chromosomes, the ‘RGB Profile Plot’ plugin for ImageJ software was used. Photomicrographs from the preparations with isolated nuclei (histone post-translational modifications immunopatterns and FISH) were taken using an Olympus FV-1000 confocal microscope and then processed using MBF ImageJ (NIH, US).

### Silver staining procedure

Silver staining was performed according to Hizume *et al*.^50^. The meristems squashed on slides were washed in borate buffer (0.01 M Na_2_B_4_O_7_, pH 9.2) for 20 min and air-dried. Then, a freshly prepared 50% aqueous silver nitrate solution was applied to each slide. The preparations were covered with a nylon tissue meshes and incubated at 42 °C in a humid chamber for 20–40 min. After three washes in distilled water, slides were air-dried and mounted in DPX mountant for histology (Sigma).
